# Surgical Therapy for Peri‐Implantitis Using Deproteinised Bovine Bone Mineral With 10% Collagen, Enamel Matrix Derivative and Doxycycline: A Prospective Cohort 10‐Year Implant Study

**DOI:** 10.1111/jcpe.70175

**Published:** 2026-07-23

**Authors:** Faustino Mercado, Saso Ivanovski

**Affiliations:** ^1^ Faculty of Dentistry University of Otago Dunedin Otago New Zealand; ^2^ Sir John Walsh Research Institute University of Otago Dunedin Otago New Zealand; ^3^ School of Dentistry University of Queensland Brisbane Australia

**Keywords:** Deproteinised bovine bone with 10% collagen, enamel matrix derivative, implant survival, peri‐implantitis, success criterion

## Abstract

**Aim:**

To assess 10‐year implant survival following surgical management of peri‐implantitis using deproteinised bovine bone mineral with 10% collagen (DBBMC), enamel matrix derivative (EMD) and doxycycline powder.

**Materials and Methods:**

The original population comprised 30 non‐smoking, periodontally stable patients (mean age = 44.9 ± 11 years; 64% female; full‐mouth plaque and bleeding score < 20%, no bleeding residual periodontal pockets ≥ 4 mm), each with a single peri‐implantitis‐affected implant (bleeding on probing, probing depth > 6 mm, ≥ 20% radiographic bone loss, implants in function ≥ 2 years). The surgical procedure involved implant surface decontamination with 24% EDTA followed by defect fill with DBBMC/EMD/doxycycline mixture. Patients underwent individualised supportive peri‐implant therapy (SPIT) comprising regular clinical monitoring, professional cleaning and antimicrobial protocols for 10 years.

**Results:**

Twenty‐nine of the 30 implants survived (96.6% survival rate). Mean probing depth decreased from 8.90 ± 1.9 mm to 3.06 ± 0.98 mm; radiographic bone loss improved from 57% ± 16.5% to 13% ± 2.5%. During 10‐year SPIT, 12 implants required adjunctive azithromycin. Fifty‐eight percent achieved the successful treatment outcome criterion (SOTC: probing depth < 5 mm, no bone loss 10% from baseline, absence of bleeding on probing/suppuration and recession < 0.5 mm anterior/1.5 mm posterior implants).

**Conclusion:**

Surgical management of peri‐implantitis combined with DBBMC/EMD/doxycycline and personalised SPIT achieved high long‐term implant survival and stable clinical outcomes over 10 years.

## 1 Introduction

Peri‐implantitis is an inflammatory disease affecting tissues surrounding osseointegrated dental implants, characterised by progressive marginal bone loss and bleeding or suppuration on probing (Schwarz et al. 2018; Zitzmann and Berglundh 2008). Recent systematic review data reveal a peri‐implantitis prevalence of 19.53% (95% CI 12.87–26.19) at the patient level and 12.53% (95% CI 11.67–13.39) at the implant level (Diaz et al. 2022), with rates increasing when diagnostic thresholds are lowered—for example, using bleeding pockets exceeding 4 mm rather than 6 mm (Koldsland et al. 2010). As dental implant use continues to expand, the clinical burden of peri‐implant disease has correspondingly increased, making early detection and effective management essential for preserving implant function and minimising the need for complex revision procedures.

Non‐surgical management has shown limited efficacy for peri‐implantitis (Polymeri et al. 2022; Faggion Jr et al. 2014), although adjunctive systemic antibiotics have been associated with improved disease resolution in 56%–67% of cases (Liñares et al. 2024; Leira et al. 2026). Consequently, diverse surgical approaches, including regenerative and resective techniques—have been developed, yielding variable outcomes (Roccuzzo et al. 2020; Roccuzzo et al. 2017; Mercado et al. 2018; Roos‐Jansåker et al. 2007; Schwarz et al. 2017). Surgical success is influenced by multiple factors: implant surface characteristics (Doornewaard et al. 2017; Carcuac et al. 2020; Roccuzzo et al. 2011), bone defect morphology (Aghazadeh et al. 2020; Schwarz et al. 2010), patient's systemic health including diabetes and smoking status (Renvert et al. 2014; Zhu et al. 2024; Trullenque‐Eriksson et al. 2025), bone substitute materials and growth factors or collagen membranes (Isler et al. 2025) and the soft‐tissue phenotype (Isler et al. 2024; Sculean et al. 2019).

This study's favourable outcomes were substantially influenced by rigorous case selection restricted to crater‐like osseous defects. This morphological homogeneity represents a critical strength: crater‐shaped defects feature intact bony margins providing structural scaffolding for optimal graft positioning, facilitating haemostatic stability and preserving the provisional blood clot matrix essential for osteogenic differentiation. Published evidence supports this selective approach, demonstrating that contained defect morphologies—particularly crater and three‐wall configurations—yield significantly improved probing depth reduction and clinical attachment gains compared to non‐contained defects (Schwarz et al. 2018; Mordini et al. 2021; Noelken and Al‐Nawas 2023).

Baseline probing pocket depth (PPD) represents the most significant predictor of surgical therapy outcomes, with deeper initial pockets reducing the probability of achieving pocket resolution (PPD ≤ 5 mm) at 12 months (Ichioka et al. 2023). Smoking status significantly influences treatment response, with smokers showing persistently elevated residual pocket depths compared to non‐smokers (Ichioka et al. 2023). Despite diverse risk factors, consensus exists regarding the critical importance of patient oral hygiene and long‐term, individualised supportive peri‐implant and periodontal therapy (SPIT) for successful peri‐implant outcomes (Atieh et al. 2021; Heitz‐Mayfield et al. 2018; Mercado et al. 2018). Limited data provide robust 5–10‐year follow‐up for surgically treated peri‐implantitis (La Monaca et al. 2025; Froum 2022; Roccuzzo et al. 2020; Bianchini et al. 2019; Berglundh et al. 2018; Heitz‐Mayfield et al. 2018). Existing studies report variable implant survival rates, typically exceeding 80%–90% at 5 years but declining with extended follow‐up (Froum 2022; Roccuzzo et al. 2020; Bianchini et al. 2019; Berglundh et al. 2018; Heitz‐Mayfield et al. 2018; Roccuzzo et al. 2017). Variability in surgical techniques, patient selection, maintenance protocols and outcome definitions complicates cross‐study comparison.

Enamel matrix derivative (EMD) possesses dual biological mechanisms: antimicrobial properties (Arweiler et al. 2002; Sculean et al. 2001) and osteogenic potential (Mercado et al. 2021; Stout et al. 2014; Prata et al. 2007), both relevant to peri‐implantitis management. Doxycycline provides multifactorial therapeutic effects—antimicrobial activity, matrix metalloproteinase inhibition and anti‐collagenase properties—demonstrating clinical efficacy as adjunctive therapy (D'Orto and Polizzi 2025; Golub et al. 1990). The rational combination of these agents may enhance peri‐implant disease management through bacterial load reduction and host inflammatory modulation.

This study presents 10‐year implant survival outcomes following surgical reparative therapy integrating deproteinised bovine bone mineral with EMD and doxycycline, evaluating clinical parameters and radiographic outcomes in patients receiving long‐term SPIT.

## 2 Materials and Methods

### 2.1 Patient Selection

The treatment protocol has been comprehensively outlined in prior publications addressing the 3‐year treatment outcomes (Mercado et al. 2018). In summary, the initial cohort consisted of 30 consecutive individuals who were referred for the management of peri‐implantitis at the Western Sydney Specialist Periodontal Practice during the period from January 2009 to January 2012. The ages of the participants varied from 20 to 70 years (Supporting Information S1).

The criteria for inclusion in the study were established as follows:

1. Participants were required to be systemically healthy and non‐smokers.
2. Participants must demonstrate adequate oral hygiene practices, with full‐mouth plaque score of < 20%.
3. Participants needed to have completed a pre‐surgical phase of periodontal treatment, which involved full‐mouth ultrasonic debridement and manual curettage, resulting in a full‐mouth bleeding score (FMBS) of < 20%, with no residual bleeding periodontal pockets > 4 mm.
4. Only one implant could be affected by peri‐implantitis, characterised by a ‘crater‐like’ or circumferential defect.
5. The affected implant must demonstrate the following diagnostic parameters: (a) a pocket depth ≥ 6 mm with evidence of bleeding on probing (BOP) or purulent exudate; (b) radiographic evidence of circumferential alveolar bone loss ≥ 20% relative to the implant length; and (c) a minimum functional duration of ≥ 2 years in situ.

The following criteria were established for exclusion from the study:

1. Individuals diagnosed with uncontrolled diabetes.
2. Individuals currently prescribed bisphosphonate medications.
3. Pregnant or lactating women.
4. Individuals exhibiting confirmed mobility of dental implants.
5. Dental implants that are not optimally positioned.

This observational study was conducted in accordance with the revised principles of the Declaration of Helsinki and Good Clinical Practice Guidelines. The study protocol was approved by the Griffith University Research Ethics Committee (GUHREC 2016/924). All patients received a comprehensive explanation of the surgical procedures and study objectives before consenting to participate. Informed consent forms were signed before inclusion in the study. The project is registered with the Australian and New Zealand Clinical Trials Registry (ACTRN12619000062123). The study's findings are reported in accordance with the EQUATOR guidelines for cohort studies (STROBE), available at https://www.equator‐network.org.

### 2.2 Surgical Phase

**FIGURE 1:**
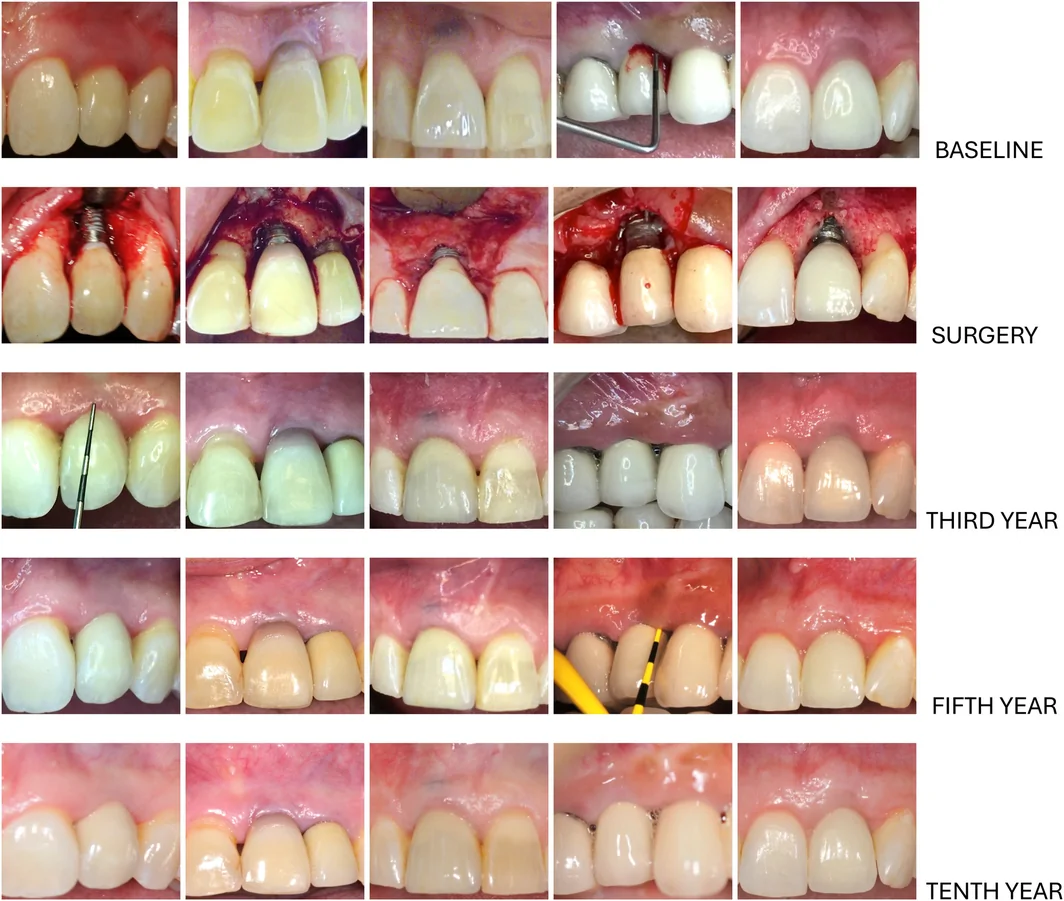
Dental implants with connective tissue graft used as part of the surgical therapy of peri‐implantitis protocol from baseline to 10th year.

The pre‐surgical protocol is detailed in a previous publication (Mercado et al. 2018). Briefly, all surgeries were performed under local anaesthesia (lidocaine HCL 2% with 1:100,000 epinephrine) and oral sedation (diazepam 5–10 mg, administered 1 h earlier). After flap elevation and removal of granulation tissue, exposed implant threads were debrided with a low‐power ultrasonic scaler (Cavitron SPS, Dentsply, York, PA, USA). The implant surface was dried with gauze, treated with 24% ethylenediamine tetraacetic acid (EDTA, Prefgel, Switzerland) for 2 min and rinsed with saline. Deproteinised bovine bone mineral with 10% collagen (DBBMC) (Bio‐Oss Collagen, Geistlich, Switzerland) was mixed with 0.35 mL enamel matrix derivative (EMD) (EMDOGAIN, Straumann, Basel, Switzerland) for 15 min in a sterile dish. One capsule of doxycycline 100 mg was added and blended until homogeneous. This mixture was applied to all exposed threads. Primary closure was achieved using a coronally advanced flap (CAF). Connective tissue graft (CTG) was used as needed, particularly when keratinised gingiva was less than 1 mm or the defect was in the anterior maxilla (Figure 1). CTG was applied to six maxillary anterior and two mandibular posterior implants. Only resorbable sutures (3–0, Vicryl, Johnson & Johnson, Diegem, Belgium) were used. Routine post‐operative instructions were followed as detailed in a previous publication (Mercado et al. 2018).

### 2.3 Supportive Periodontal and Implant Therapy

**FIGURE 2:**
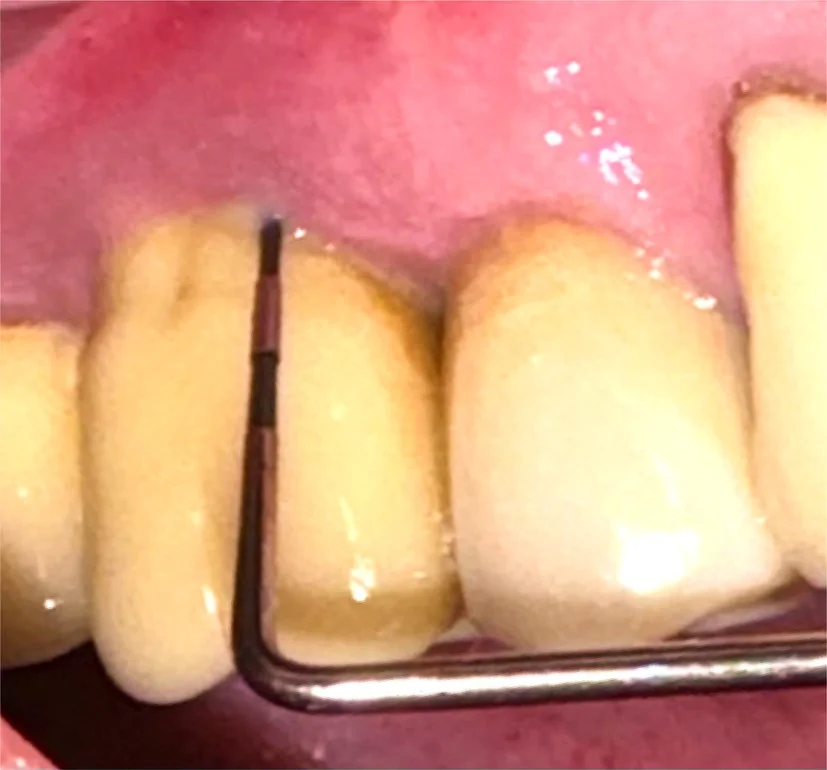
Dental implant exhibiting suppuration during supportive periodontal and implant therapy (SPIT).

All patients received an individualised SPIT programme customised to their unique requirements. Each SPIT appointment comprised oral hygiene measures and comprehensive debridement of the entire mouth, including the treated implant, using a Cavitron SPS (Dentsply, York, PA, USA). Most implants underwent evaluations every 4–6 months. In instances of bleeding on probing (BOP), additional manual curettage, administered with or without local anaesthesia using Gracey curettes (Hu‐Friedy, Chicago, USA), was performed on the affected implant or dentition. If suppuration was observed on an implant during a visit, the patient was prescribed azithromycin 500 mg (three tablets) over 3 days (Figure 2). The number of implants treated with supplementary systemic azithromycin was documented. No patients required additional surgical intervention during the 10‐year observation period.

### 2.4 Clinical Measurements

**FIGURE 3:**
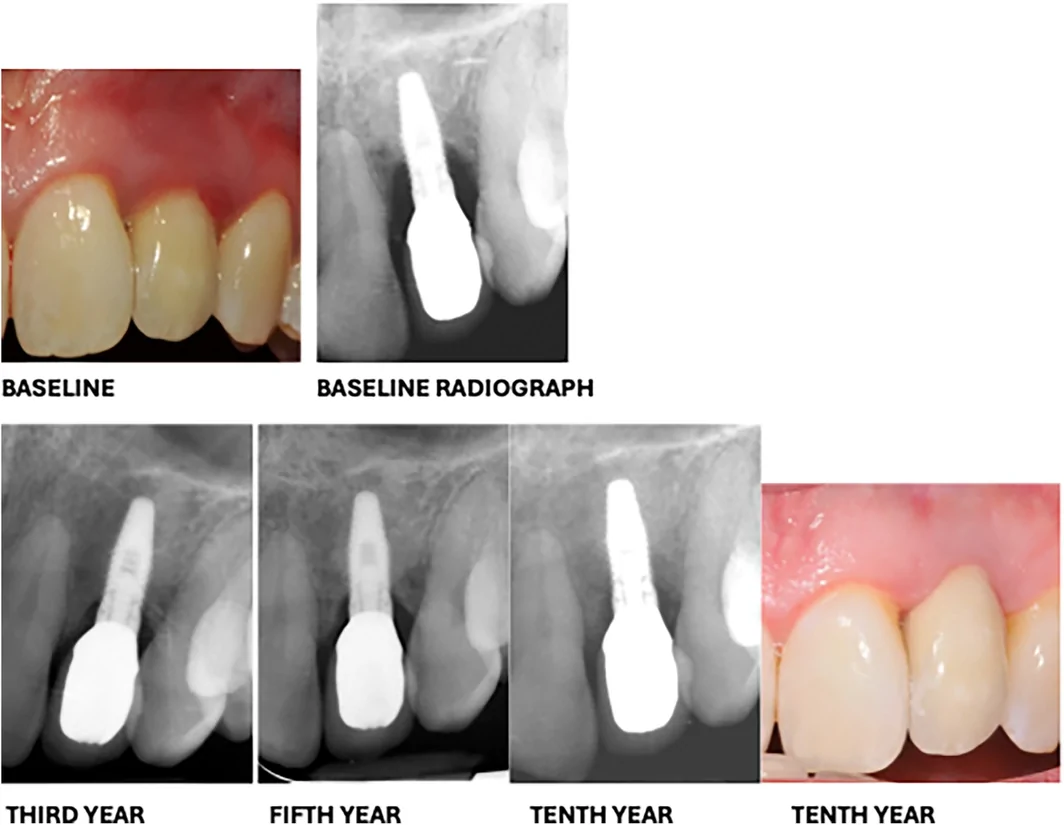
Radiographs acquired at the baseline assessment, as well as those recorded during the evaluations conducted in the 3rd, 5th and 10th years.

Clinical and radiographic parameters (recession, probing depth, buccal keratinised tissue, peri‐apical radiographs) were collected as described in a previous paper (Mercado et al. 2018). Clinical parameters recorded at baseline, 2nd year, 3rd year, 5th year and 10th year were tabulated for analysis (Figures 3 and S3). An oral health therapist (OHT) with more than 10 years of experience, who was not involved in the surgical procedure (V.S.), documented all clinical and radiographic parameters using ×3.5 magnification. Additionally, the presence of dental plaque (Pl), BOP and suppuration was recorded. To ensure intra‐examiner reproducibility, a calibration exercise was conducted involving full‐mouth charting of five patients over three consecutive weeks, confirming measurement accuracy within 0.5 mm.

### 2.5 Successful Treatment Outcome Criterion (STOC)

A composite criterion was established to evaluate the successful treatment of implants and the need for any additional surgical intervention (Heitz‐Mayfield and Mombelli 2014; Mercado et al. 2018). The success criteria proposed for the 10th year are as follows:

1. An implant exhibiting the deepest probing depth (PD) of less than 5 mm.
2. No further bone loss exceeding 10% when compared to baseline.
3. Absence of BOP or suppuration.
4. Recession of < 0.5 mm for anterior implants and < 1.5 mm for posterior implants.

These criteria provide a rigorous framework for assessing implant success and overall patient outcomes.

### 2.6 Statistical Analyses

A paired *t*‐test was employed to evaluate the differences in clinical parameters (PD, REC, bone level) at baseline, as well as at the 2nd, 3rd, 5th, and 10th years. A *p*‐value of < 0.01 was deemed statistically significant for this analysis.

## 3 Results

### 3.1 Patient and Implant Attributes

Of the 30 enrolled patients and implants, 29 completed the 10‐year follow‐up. The cohort had a 2:1 female/male ratio and a mean age of 44.9 years (±11). All participants maintained good oral hygiene, with FMPS and FMBS < 20% (Table 1, Supporting Information S2).

**TABLE 1:** Patient and implant distributions at baseline.

Mean age (year) of patients	Male %	Female %	Mean age (year) of implants
44.9 (±11)	36	64	9 (±3.8)
Location of Implants (%)			
Mandibular posterior	40		
Mandibular anterior	10		
Maxillary posterior	16.6		
Maxillary anterior	33.3		
Types of Implants treated (%)			
Branemark TiUnite	46.66		
Astra Tech	26.66		
Straumann	10		
Others	16.66		

### 3.2 | Clinical Parameters

The surgical reparative approach proved effective against moderate to advanced peri‐implantitis. Mean bone loss fell from 6.92 mm (±1.26; 57% ±16.50 of marginal bone level) at baseline to 2.62 mm (±0.80; 14.8% ±8.2) post treatment (*p* < 0.01). Stability held at 2.60 mm (±0.73; 14.5% ±7.30) at 3 years, 2.73 mm (±0.67; 12.3% ±2.3) at 5 years and 2.78 mm (±0.56; 12.86% ±2.47) at 10 years, with significance maintained throughout (*p* < 0.01; Table 2).

**TABLE 2:** Clinical parameters.

Parameters	Baseline	2nd year	3rd year	5th year	10th year
Bone loss (mm)	6.92 ± 1.26	2.62 ± 0.80*	2.60 ± 0.73*	2.73 ± 0.67*	2.78 ± 0.56*
Bone loss %	57 ± 16.50	14.8 ± 8.2*	14.5 ± 7.30*	12.30 ± 2.3*	12.86 ± 2.47*
Probing depth (mm)	8.90 ± 1.90	3.50 ± 0.50*	3.5 ± 0.50*	3.20 ± 1.12*	3.06 ± 0.98*
Mid‐buccal recession (mm)	0.16 ± 0.14	0.15 ± 0.12	0.22 ± 0.19	0.20 ± 0.42	0.48 ± 0.96
Keratinised tissue (mm)	1.79 ± 0 0.79	1.90 ± 0.56	1.93 ± 0.54	1.50 ± 0.46	1.48 ± 0.53

*Note:* Number of implants = Baseline to 2nd year, 3rd year and 5th year = 30, *N* = 10th year 29, *p < 0.01 statistically significant from baseline.

Mean probing depth (PD) dropped from 8.90 mm (±1.90) at baseline to 3.50 mm (±0.50) by 2 and 3 years (*p* < 0.01), then to 3.20 mm (±1.12) at 5 years and 3.06 mm (±0.98) at 10 years. Changes between 3, 5 and 10 were non‐significant, confirming clinical stability from 3 years onward (Table 2).

Buccal recession was minimal at baseline (0.16 ± 0.14 mm) and stable through Year 2 (0.15 ± 0.12 mm), rising modestly to 0.22 (±0.19), 0.20 (±0.42) and 0.48 (±0.96) mm at years 3, 5 and 10, respectively. Differences across time points were non‐significant.

At baseline, 100% of implants showed BOP and suppuration on probing (SOP). FMBS improved from 70% (±10) at baseline to 18% (±8), 15% (±9), 17% (±8) and 16% (±8) at years 2, 3, 5 and 10, with FMPS consistently below 20%. Suppuration recurred in 12 implants across visits (six maxillary and six mandibular posterior). These cases were managed with subgingival ultrasonic debridement and curettage under local anaesthesia, followed by azithromycin (500 mg daily for 3 days). Two‐week reassessment confirmed suppuration resolution, reduced BOP and decreased PD in most implants.

Of the 30 implants, 29 remained functional at 10 years (96.66% survival); 1 mandibular posterior implant (implant 36) lost integration by Year 8 and was removed (Figure S4). Clinical parameters, bone level, PD, BOP and recession showed no significant changes between years 2, 3, 5 and 10 in surviving implants.

Under STOC (PD < 5 mm, no further bone loss 10%, no BOP/SOP, recession ≤ 0.5 mm anterior/1.5 mm posterior), 58% of implants succeeded at Year 10 while 40% failed. The causes of failure were persistent BOP in five implants (17.24%), further interproximal bone loss in four (13.79%), anterior recession > 0.5 mm in two (6.89%) and posterior recession > 1.5 mm in one (3.44%) (Table 3).

**TABLE 3:** Success criterion after 10 years.

Success criterion	Success rate after 10 years (*n* = 29/30 implants)
Probing depth > 5 mm	0
Further bones loss greater than 10% when compared to baseline bone level	4
+BOP/suppuration	5
Recession	
Anterior implants (≥ 0.5 mm)	2
Posterior implants (≥ 1.5 mm)	1
Percentage of treatment failure	41.37% (12/29)
Percentage of treatment success	58.62% (17/29)

## 4 Discussion

This prospective cohort study evaluated 30 consecutive patients with peri‐implantitis treated using a standardised surgical protocol combining DBBMC, EMD and doxycycline powder, followed for 10 years. All patients received individualised SPIT throughout the observation period.

Supportive care is fundamental to long‐term implant stability (Costa et al. 2023). Prospective data show that patients declining structured preventive maintenance experienced markedly elevated peri‐implantitis development (43.9% vs. 18.0% in those adhering to scheduled interventions) (Costa et al. 2012). An 11‐year longitudinal assessment corroborated these findings, showing that consistent SPIT adherence substantially attenuated peri‐implantitis incidence (11.1% regular adherents vs. 37.5% irregular adherents), with elevated bleeding indices and increased PDs as significant prognostic indicators (Costa et al. 2023). This study showed sustained 96.6% implant retention across 10 years through systematic 4–6 month clinical reassessment with targeted antimicrobial intervention, underscoring the necessity of protocolised supportive care.

Treatment outcomes show progressive efficacy decline over extended follow‐up, with patient success diminishing from approximately 79% at 1 year to 63% by 5 years, while implant retention deteriorates from 100% initially to 55%–80% after a decade, with variation largely attributable to implant surface topography (Heitz‐Mayfield et al. 2018; Roccuzzo et al. 2020). Even with contemporary surgical debridement and bone regeneration protocols, complete disease resolution—defined by the absence of bleeding upon gentle probing—remains elusive, occurring in merely 23%–42% of implants, with recurrent inflammatory episodes developing in 30%–35% of treated patients within comparable timeframes (Isler et al. 2025; Roccuzzo et al. 2020). Bone‐substitute scaffolds with or without absorbable membranes reveal equivalent clinical and radiographic improvements, including mean PD reductions of 3.0–3.3 mm, indicating that procedural technique matters less than adherence to comprehensive care protocols (Roos‐Jansåker et al. 2014).

Froum et al. (2015) reported that 170 implants followed for 10 years frequently required multiple additional surgeries. Conversely, no patients in this cohort required re‐surgery. The 58% success rate (SOTC) aligns with previously reported 50%–60% rates using similar criteria (Ramanauskaite et al. 2023; Froum 2022; Heitz‐Mayfield et al. 2018; Carcuac et al. 2020). Absence of re‐intervention may reflect patient selection (crater‐like defects only) and adjunctive EMD and doxycycline use, which enhance healing and bone stability through antimicrobial and host‐modulating properties. Favourable outcomes also reflect strict inclusion criteria: systemically healthy, non‐smoking patients with good oral hygiene and plaque/bleeding scores below 20%.

Roccuzzo et al. (2016, 2017, 2020) employed identical decontamination (24% EDTA) and xenograft material across 5, 7 and 10‐year follow‐ups. This study's 5‐year findings demonstrated comparable clinical attachment: PPD 3.20 mm (±1.12) versus 3.3 mm (±0.9), crestal bone resorption 2.73 mm (±0.67) versus 2.90 mm (±1.10), with negligible soft‐tissue recession (0.16 ± 0.14). At 10 years, the maintained favourable parameters included PD 3.06 mm (±0.98), bone resorption 2.78 mm (±0.56) and implant retention 96.66%. Roccuzzo et al. (2020) documented substantially lower success with demineralised bone matrix and collagen alone (67% retention, 42% success). Isler et al. (2025), using concentrated growth factor with membranes, identified deepest pocket depth as a significant prognostic indicator (*p* = 0.008), yielding 81.25%–88.8% retention and 23.1%–35.3% success at 7 years. This EMD/doxycycline‐augmented xenograft protocol compares favourably with other long‐term regenerative studies.

During 10‐year follow‐up, 12 implants that showed suppuration were successfully managed with mechanical debridement and short‐course azithromycin, resolving suppuration and improving probing depths. A systematic review by Antonoglou et al. (2025) of seven studies encompassing 595 patients and 1388 implants found that systemic antibiotics significantly improved short‐term outcomes: higher success rates (OR = 2.33; 95% CI: 1.29–4.21), modest bone gain (MD = 0.37 mm; 95% CI: −0.68 to −0.07), improved stability (OR = 2.73; 95% CI: 1.50–4.99) and reduced bleeding (OR = 0.49) and suppuration (OR = 0.33), with benefits more pronounced in modified‐surface implants during the first 1–2 years. However, Hallström et al. (2017) suggested that azithromycin might not significantly enhance outcomes with open‐flap debridement alone.

PD correlates with sub‐implant microbiota characteristics, suggesting their utility as biomarkers for disease progression (Monje and Salvi 2024). Contemporary evidence shows that combined surgical intervention, regenerative modalities and systematic supportive therapy substantially enhance implant survival (Heitz‐Mayfield et al. 2018). However, achieving complete peri‐mucosal health remains challenging. This cohort's analysis showed that 6‐month maintenance intervals were associated with 50% recurrent bleeding, reducing to 20% with 4‐month intervals (Mercado et al. 2018), indicating heightened inflammatory recurrence susceptibility when intervals exceed 4 months. Despite adherence to 4–6‐monthly protocols, 20%–30% exhibited persistent bleeding, suggesting distinct patient phenotypes predisposing towards post‐operative relapse. Radiographic assessment at 5 and 10 years revealed stable osseous levels, with some implants with bleeding/suppuration demonstrating negligible bone loss. BOP carries only 24.1% predictive probability for peri‐implantitis diagnosis (Hashim et al. 2018), although chronic mild bleeding patterns correlate more strongly with progressive bone loss than isolated severe episodes (de Tapia et al. 2026). Single bleeding sites may reflect probe‐induced trauma rather than disease (Monje and Salvi 2024). Therefore, BOP should integrate with visible inflammation, PD and radiographic bone loss before establishing diagnosis.

Baseline PPD significantly predicts outcomes (Ichioka et al. 2023). This cohort was treated for substantially more advanced disease (baseline 8.90 ± 1.90 mm vs. Ichioka's 4.8 ± 1.8 mm), yet achieved superior reduction to 3.50 mm by Year 3 and maintained it at 3.06 mm (±0.98) at 10 years, with a minimal soft‐tissue recession of 0.48 mm (±0.96) versus Ichioka's 1.0 mm at 12 months. However, a 120‐patient multi‐centre trial found no significant difference between access flap surgery with or without bone grafting (Alibegovic et al. 2025), suggesting that discrepancies reflect defect morphology, surgical protocols or patient factors.

Connective tissue graft (CTG) augmentation in eight implants with < 1 mm keratinised mucosa or thin phenotype showed comparable baseline severity (bone loss 6.9 ± 1.21 mm; PD 8.90 ± 1.8 mm). By Year 2, substantial improvement occurred (bone loss 2.5 ± 0.60 mm; PD 3.40 ± 0.40 mm), stabilising through year 10 (bone loss 2.625 ± 0.48 mm; PD 2.81 ± 0.57 mm, comparable to non‐CTG: PD 3 ± 0.79 mm; bone loss 2.95 ± 0.76 mm). CTG significantly increased keratinised tissue (1.93 ± 0.17 mm versus 1.34 ± 0.39 mm; *p* = 0.0003) and reduced recession (0.00 vs. 0.56 ± 0.50 mm; *p* = 0.0028) (Table 4). All CTG‐augmented implants achieved SOTC success, aligning with American Academy of Periodontology and Academy of Osseointegration recommendations (Wang et al. 2025; Fiorellini et al. 2025).

**TABLE 4:** CTG group versus non‐CTG group—Clinical parameters from baseline to 10th year.

	Baseline PD (mm)	Baseline bone level (mm)	Baseline recession (mm)	Baseline KT (mm)	10th year PD (mm)	10th year bone level (mm)	10th year recession (mm)	10th year KT (mm)
CTG Group (8 implants)	8.90 ± 1.8	6.9 ± 1.21 (58% ± 12.4%)	0.15 ± 0.25	1.7 ± 0.65	2.81 ± 0.57	2.625 ± 0.48 (12.75% ± 1.92%)	0.00 ± 0.00^a^	1.93 ± 0.17^a^
Non‐CTG group (21 implants)	8.31 ± 0.92	7.27 ± 0.91 (54.63 ± 6.034)	0.43 ± 0.45	0.954 ± 0.208	3 ± 0.79	2.95 ± 0.76 (13.13% ± 2.59%)	0.59 ± 0.50^a^	1.34 ±0.39 mm^a^

^a^ Statistically significant (*p* < 0.001), when compared to non‐CTG group at 10th year.

One implant failed at Year 8 because of osseointegration loss, emphasising the importance of ongoing occlusal assessment (Mojaver et al. 2025; Tallarico et al. 2025). Surgical and maintenance procedures performed without prosthesis removal reduced treatment time and cost, as supported by Astolfi et al. (2021) finding no significant difference between removal and retention. However, evidence suggests that submerged healing may improve outcomes (Wen et al. 2024), with recent consensus recommending this approach when feasible (Fiorellini et al. 2025).

## 5 Conclusion

This study demonstrates that reconstructive surgical therapy achieves approximately 60% clinical success with long‐term results. Limitations include lack of concurrent controls preventing individual therapeutic component evaluation, limited cohort size and single‐institution design restricting generalisability. Rigorously designed multi‐centre randomised controlled trials with extended follow‐up are essential to establish definitive efficacy of regenerative biomaterials and complementary interventions in comprehensive peri‐implantitis treatment.

## Author Contributions

F.M. conceived the ideas, performed the surgical procedures, analysed the data and led the writing. S.I. conceived the ideas, led the writing and edited the manuscript.

## Funding

FM was partly supported by a scholarship from the National Health and Medical Research Council Australia.

## Conflicts of Interest

The authors declare no conflicts of interest.

## Supporting information


**Supporting Information S1:** Additional information on materials and methods detailing patient selection criteria, surgical protocol and supportive periodontal and implant therapy (SPIT) for the 10‐year prospective cohort study.

**Supporting Information S2:** Additional results detailing patient and implant attributes and clinical parameters, including bone level changes, probing depth, recession, bleeding on probing and suppuration and implant survival outcomes over the 10‐year follow‐up period.

**Figure S3:** Probing depth examination during supportive periodontal and implant therapy (SPIT).

**Figure S4:** Failure of Implant 36, which resulted from a loss of integration on the 8th year of supportive periodontal and implant therapy (SPIT).

## Data Availability

The data supporting the findings of this study are available within the article, as detailed in the relevant tables presented in the manuscript.
