# Supplementary material for: Surgical Therapy for Peri‐Implantitis Using Deproteinised Bovine Bone Mineral With 10% Collagen, Enamel Matrix Derivative and Doxycycline: A Prospective Cohort 10‐Year Implant Study
Source: J Clin Periodontol. 2026 Jul 23;53(10):1575–84. doi: 10.1111/jcpe.70175 (PMC13581473; doi:10.1111/jcpe.70175)
Supplement: Supplementary file 2 [file JCPE-53-1575-s002.docx]

SUPPLEMENTARY Figure S3

Figure 3. Probing depth examination during supportive periodontal and implant

therapy (SPIT)
