# Supplementary material for: Surgical Therapy for Peri‐Implantitis Using Deproteinised Bovine Bone Mineral With 10% Collagen, Enamel Matrix Derivative and Doxycycline: A Prospective Cohort 10‐Year Implant Study
Source: J Clin Periodontol. 2026 Jul 23;53(10):1575–84. doi: 10.1111/jcpe.70175 (PMC13581473; doi:10.1111/jcpe.70175)
Supplement: Supplementary file 3 [file JCPE-53-1575-s003.docx]

Supplementary Material S1

MATERIALS AND METHODS Additional Information

**2.0 Materials and Methods**

2.1 Patient Selection

The treatment protocol has been comprehensively outlined in prior publications addressing the three-year treatment outcomes (Mercado et al., 2018). In summary, the initial cohort consisted of 30 consecutive individuals who were referred for the management of peri-implantitis at the Western Sydney Specialist Periodontal Practice during the period from January 2009 to January 2012. The ages of the participants varied from 20 to 70 years.

The criteria for inclusion in the study were established as follows:

1. Participants were required to be systemically healthy and non-smokers.

2. Participants must demonstrate adequate oral hygiene practices, with Full Mouth Plaque score of less than 20%.

3. Participants needed to have completed a pre-surgical phase of periodontal treatment, which involved full-mouth ultrasonic debridement and manual curettage, resulting in a Full-Mouth Bleeding Score (FMBS) of less than 20% with no residual bleeding periodontal pockets greater than 4 mm.

4. Only one implant could be affected by peri-implantitis, characterised by a “crater-like” or circumferential defect.

**5. T**he affected implant must demonstrate the following diagnostic parameters: (a) a pocket depth ≥6 mm with evidence of bleeding on probing (BOP) or purulent exudate; (b) radiographic evidence of circumferential alveolar bone loss ≥20% relative to the implant length; and (c) a minimum functional duration of ≥2 years in situ.

The following criteria were established for exclusion from the study:

1. Individuals diagnosed with uncontrolled diabetes;

2. Individuals currently prescribed bisphosphonate medications;

3. Pregnant or lactating women;

4. Individuals exhibiting confirmed mobility of dental implants;

5. Dental implants that are not optimally positioned.

This observational study was conducted in accordance with the revised principles of the Declaration of Helsinki and Good Clinical Practice Guidelines. The study protocol was approved by the Griffith University Research Ethics Committee (GUHREC 2016/924). All patients received a comprehensive explanation of the surgical procedures and study objectives before consenting to participate. Informed consent forms were signed before inclusion in the study. The project is registered with the Australian and New Zealand Clinical Trials Registry (ACTRN12619000062123). The study's findings are reported in accordance with the EQUATOR guidelines for cohort studies (STROBE), available at https://www.equator-network.org.

2.2 | Surgical Phase

The pre-surgical protocol is detailed in a previous publication (Mercado et al 2018). Briefly, all surgeries were performed under local anaesthesia (Lidocaine HCL 2% with 1:100,000 epinephrine) and oral sedation (Diazepam 5–10 mg, administered one hour prior). After flap elevation and removal of granulation tissue, exposed implant threads were debrided with a low-power ultrasonic scaler (Cavitron SPS, Dentsply, York, PA, USA). The implant surface was dried with gauze, treated with 24% ethylenediamine tetraacetic acid (EDTA, Prefgel, Switzerland) for 2 minutes, and rinsed with saline. Deproteinised bovine bone mineral with 10% collagen (DBBMC) (Bio-Oss Collagen, Geistlich, Switzerland) was mixed with 0.35 ml enamel matrix derivative (EMD) (EMDOGAIN, Straumann, Basel, Switzerland) for 15 minutes in a sterile dish. One capsule of Doxycycline 100 mg was added and blended until homogeneous. This mixture was applied to all exposed threads. Primary closure was achieved using a coronally advanced flap (CAF). Connective Tissue Graft (CTG) were used as needed, particularly when keratinised gingiva was less than 1 mm, thin phenotype or the defect was in the anterior maxilla (Figure 1). CTG were applied to six maxillary anterior and two mandibular posterior implants. Only resorbable sutures (3–0, Vicryl®, Johnson & Johnson, Diegem, Belgium) were utilised. Routine post-operative instructions were followed as detailed in a previous publication (Mercado et al 2018).

2.3 Supportive Periodontal and Implant Therapy (SPIT)

All patients received an individualised supportive periodontal and implant therapy (SPIT) program customised to their unique requirements. Each SPIT appointment comprised oral hygiene measures and comprehensive debridement of the entire mouth, including the treated implant, utilizing a Cavitron SPS (Dentsply, York, PA, USA). Most implants underwent evaluations every four to six months. In instances of bleeding on probing (BoP), additional manual curettage, administered with or without local anaesthesia using Gracey curettes (Hu-Friedy, Chicago, USA) was performed on the affected implant or dentition. If suppuration was observed on an implant during a visit, the patient was prescribed Azithromycin 500 mg (three tablets) over three days (Figure 2). The number of implants treated with supplementary systemic Azithromycin was documented. No patients required additional surgical intervention during the 10-year observation period.

2.4 Clinical Measurements

Clinical parameters (recession, probing depth, and buccal keratinised tissue) were collected as described in the previous paper (Mercado et al., 2018). Parameters recorded at baseline, 2^nd^ year, 3rd year, 5th year, and 10th year were tabulated for analysis. An Oral Health Therapist (OHT) with more than 10 years of experience, who was not involved in the surgical procedure (VS), documented all parameters using 3.5x magnification. Additionally, the presence of dental plaque (Pl), bleeding on probing (BOP), and suppuration was recorded. To ensure intra-examiner reproducibility, a calibration exercise was conducted involving full-mouth charting of five patients over three consecutive weeks, confirming measurement accuracy within 0.5 mm.

2.5 Radiographic Measurements

Radiographs were obtained using a standardised long-cone paralleling technique with an XCP-DS® X-ray holder (Chicago, USA). Images were captured at baseline and at 2, 3, 5, and 10 years. All radiographs were taken with the same Kodak 2200 intraoral X-ray unit (Trophy, Cedex 2, France) at 70 kVp, 15 mA, and 0.14 seconds. Changes in bone level (mm and % bone level) were measured by comparing the distance between the abutment-implant junction and the bone level at the mesial and distal surfaces pre-operatively and at each follow-up (Figure 3). A clinician not involved in the surgical procedure performed all radiographic measurements to ensure objectivity (VS).

2.6 Successful Treatment Outcome Criterion (STOC)

A composite criterion was established to evaluate the successful treatment of implants (Heitz-Mayfield and Mombelli, 2014, Mercado et al 2018). The success criteria proposed for the tenth year are as follows:

1. An implant exhibiting the deepest probing depth (PD) of less than 5 mm;

2. No further bone loss exceeding 10%, when compared to baseline.

3. Absence of bleeding on probing (BoP) or suppuration.

4. Recession of less than 0.5 mm for anterior implants and less than 1.5 mm for posterior implants.

These criteria provide a rigorous framework for assessing implant success and overall patient outcomes

2.7 Statistical analyses

A paired t-test was employed to evaluate the differences in clinical parameters (PD, REC, bone level) at baseline, as well as at the 3rd, 5th, and 10th years. A p-value of less than 0.01 was deemed statistically significant for this analysis.
