# Supplementary material for: Surgical Therapy for Peri‐Implantitis Using Deproteinised Bovine Bone Mineral With 10% Collagen, Enamel Matrix Derivative and Doxycycline: A Prospective Cohort 10‐Year Implant Study
Source: J Clin Periodontol. 2026 Jul 23;53(10):1575–84. doi: 10.1111/jcpe.70175 (PMC13581473; doi:10.1111/jcpe.70175)
Supplement: Supplementary file 4 [file JCPE-53-1575-s004.docx]

Supplementary Material S2

**3.0 Results**

3.1 | Patient and implant attributes

From an initial cohort of 30 patients and implants, 29 patients successfully completed a ten-year follow-up study. The demographic analysis revealed a female-to-male ratio of 2:1, with a mean age of 44.9 years (± 11). Notably, all participants maintained good oral hygiene, with Full Mouth Plaque Scores (FMPS) and Full Mouth Bleeding Scores (FMBS) both recorded at less than 20% (see Table 1).

3.2 | Clinical Parameters

3.2.1 Bone Level Changes

The surgical reparative approach demonstrated significant efficacy in treating moderate to advanced peri-implantitis. At baseline, patients presented with substantial bone loss, averaging 6.92 mm (±1.26), equivalent to 57% (±16.50) of the marginal bone level. Following treatment, bone loss was markedly reduced to 2.62 mm (±0.80), or 14.8% (±8.2), a statistically significant improvement (p < 0.01). This improvement was sustained throughout the follow-up period. At three years post-treatment, bone levels remained favourable at 2.60 mm (±0.73), or 14.5% (±7.30). Long-term stability was confirmed at five years (2.73 mm (±0.67), or 12.3% (±2.3)] and ten years (2.78 mm (±0.56), or 12.86% (±2.47)], with statistical significance maintained at all time points (p < 0.01) (Table 2).

3.2.2 Probing Depth

Peri-implant probing depth (PD) served as a key clinical indicator of disease resolution. At baseline assessment, mean PD was markedly elevated at 8.90 mm (±1.90), consistent with advanced peri-implantitis. Following treatment, PD decreased significantly to 3.50 mm (±0.50) by years two and three (p < 0.01). The reduction achieved by year three was sustained through the remainder of the study period. At five years, PD was 3.20 mm (±1.12), and by year ten, it was 3.06 mm (±0.98). Notably, changes in PD between the third, fifth, and tenth year assessments were not statistically significant, indicating that clinical stability was achieved by year three and maintained thereafter (Table 2).

3.2.3 Recession

Buccal soft tissue recession was monitored as an indicator of surgical morbidity. At baseline, mean buccal recession was minimal at 0.16 mm (±0.14). This remained stable at 0.15 mm (±0.12) through year two, with a slight increase to 0.22 mm (±0.19) observed by year three.

Recession measurements continued to show modest increases over the extended follow-up, measuring 0.20 mm (±0.42) at year five and 0.48 mm (±0.96) at year ten. Despite these gradual changes, differences in recession across all time points were not statistically significant.

3.2.4 Bleeding on Probing and Suppuration

At baseline, all treated implants (100%) presented with BoP and SoP. Over the ten-year study period, inflammatory control improved substantially. Full mouth bleeding scores (FMBS) and full mouth plaque scores (FMPS) decreased to less than 20% at every follow-up visit (baseline FMBS 70% ±10; year 2: 18% ±8; year 3: 15% ±9; year 5: 17% ±8; year 10: 16% ±8), demonstrating optimal oral hygiene maintenance by study participants. During the follow-up period, suppuration recurred in twelve implants at various time points (six maxillary posterior and six mandibular posterior implants) (Figure 2). These recurrent cases were managed with a standardised protocol: subgingival ultrasonic debridement and mechanical curettage using a Gracey curette (Hu-Friedy, Chicago, USA) under local anaesthesia, followed by systemic antibiotic therapy with Azithromycin (500 mg, one tablet daily for three days). Clinical reassessment performed two weeks post-treatment revealed positive therapeutic responses, with most implants demonstrating resolution of suppuration, reduction in bleeding on probing, and decreased probing depth.

3.2.5 Implant Survival and Success Treatment Outcome Criterion (STOC)

This study evaluated a total of 30 dental implants. At the 10-year follow-up, 29 implants remained functional, resulting in a survival rate of 96.66%. One mandibular posterior implant (implant 36) experienced loss of integration and became mobile by the eighth year of observation, which necessitated its removal (see Figure 4). Furthermore, there were no statistically or clinically significant improvements in the clinical parameters, including bone level, probing depth, bleeding on probing, and recession, throughout the monitoring periods at years 2, 3, 5, and 10 for all surviving implants.

With regard to the percentage of implants achieving success based on the Successful Treatment Outcome Criterion (STOC) at the 10th year following surgical therapy, 58% of the implants were deemed successful (Successful Treatment Outcome Criterion: probing depth < 5 mm, no further bone loss > 10%, no bleeding on probing/suppuration, and no recession greater than 0.5 mm for anterior implants and 1.5 mm for posterior implants). In contrast, 40% of the treated implants failed to meet the success criteria. The predominant causes of failure to meet the STOC parameters at 10 years post-treatment included the persistence of bleeding on probing (5 implants, 17.24%) and radiographic evidence of further interproximal bone loss (4 implants, 13.79%). Additional contributing factors to the treatment failure rate included anterior implants exhibiting gingival recession greater than 0.5 mm (2 implants, 6.89%) and posterior implants with recession exceeding 1.5 mm (1 implant, 3.44%) (Table 3).
